# Supplementary figures and images for: In vitro study of Hesperetin and Hesperidin as inhibitors of zika and chikungunya virus proteases
Source: PLoS One. 2021 Mar 4;16(3):e0246319. doi: 10.1371/journal.pone.0246319 (PMC7932080; doi:10.1371/journal.pone.0246319)

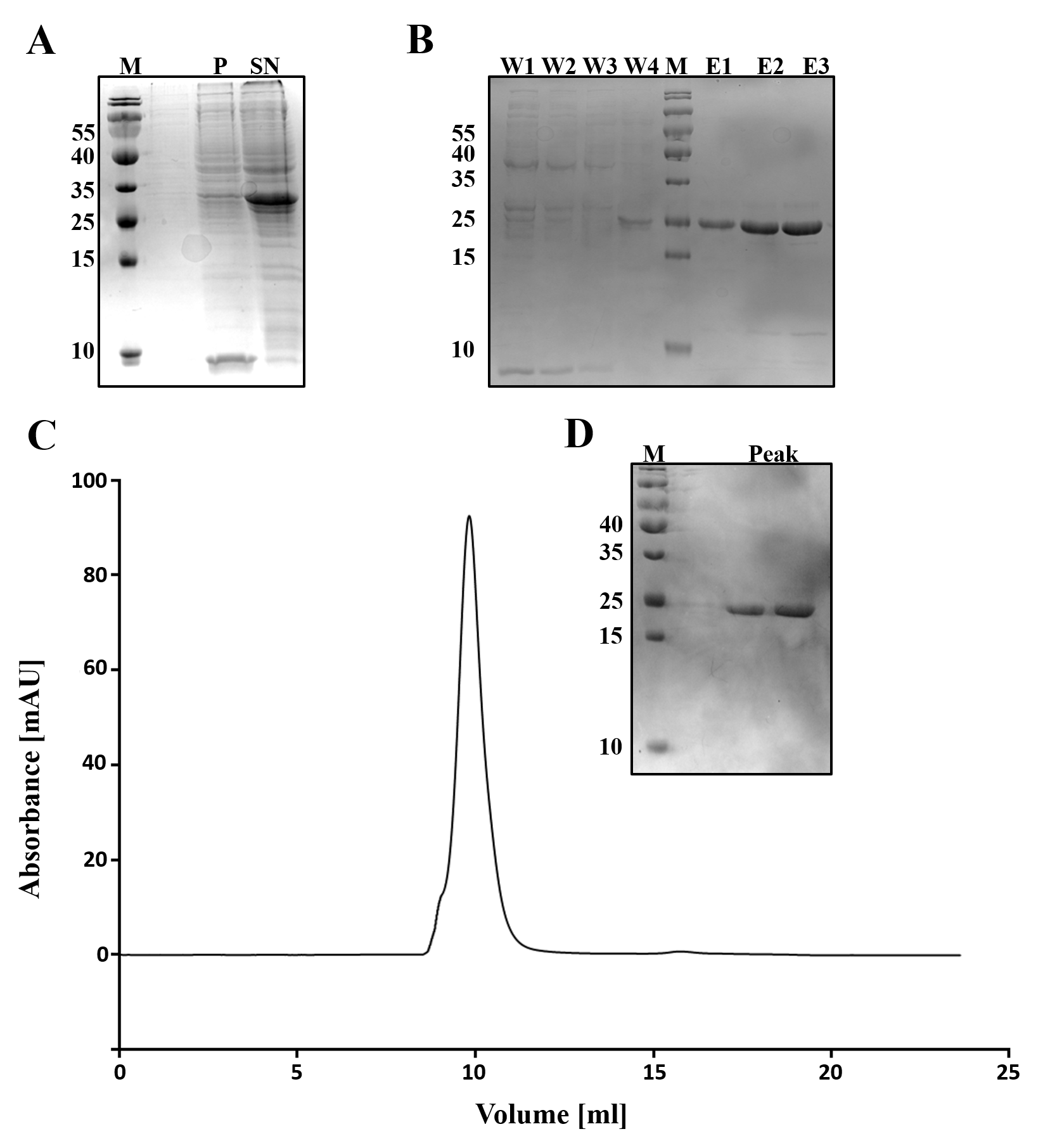

Supplement: S1 Fig — Expression and purification of ZIKV NS2B/NS3pro. The ZIKV NS2B/NS3pro construct consists of 266 amino acids with a molecular weight of 28.68 kDa. The protein presented a single band on a denaturing SDS-PAGE gel with an apparent molecular mass of approximately 30 kDa. A: SDS-PAGE analysis of ZIKV NS2B/NS3pro solubility test. M: Protein marker, P: cell pellet, SN: supernatant. B: SDS-PAGE analysis of ZIKV NS2B/NS3pro after NI-NTA purification. M: Protein marker, W1: washing step without imidazole, W2-W4: washing step with imidazole (10, 20, 40 mM), E1-E3: imidazole elution steps (80, 250, 500 mM). C: Chromatogram of size exclusion chromatography of ZIKV NS2B/NS3pro. D: SDS-PAGE of ZIKV NS2B/NS3pro after size exclusion chromatography. (TIF) [file pone.0246319.s001.tif]

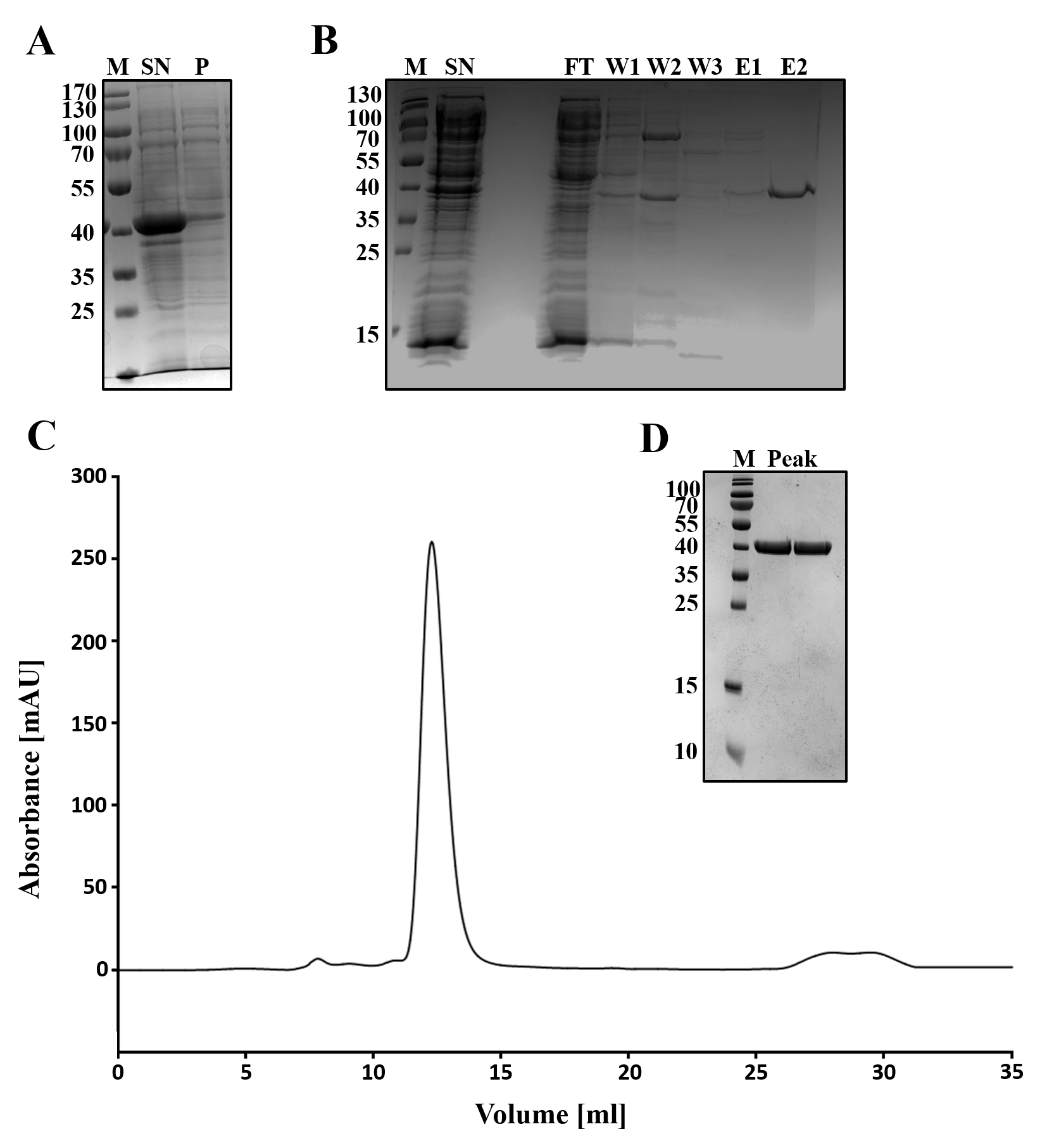

Supplement: S2 Fig — Expression, purification and CD spectrum after the preparation process of CHIKV nsP2pro. The CHIKV nsP2pro construct consists of 346 amino acids with a molecular weight of 39.38 kDa. The protein presented a single band on a denaturing SDS-PAGE gel with an apparent molecular mass of approximately 40 kDa. A: SDS-PAGE analysis of CHIKV nsP2pro solubility test. M: Protein marker, P: cell pellet, SN: supernatant. B: SDS-PAGE analysis of CHIKV nsP2pro after NI-NTA purification. M: Protein marker, W1: washing step without imidazole, W2-W3: washing step with imidazole (10, 40 mM), E1-E2: imidazole elution steps (250, 500 mM). C: Chromatogram of size exclusion chromatography of CHIKV nsP2pro. D: SDS-PAGE of CHIKV nsP2pro after size exclusion chromatography. (TIF) [file pone.0246319.s002.tif]

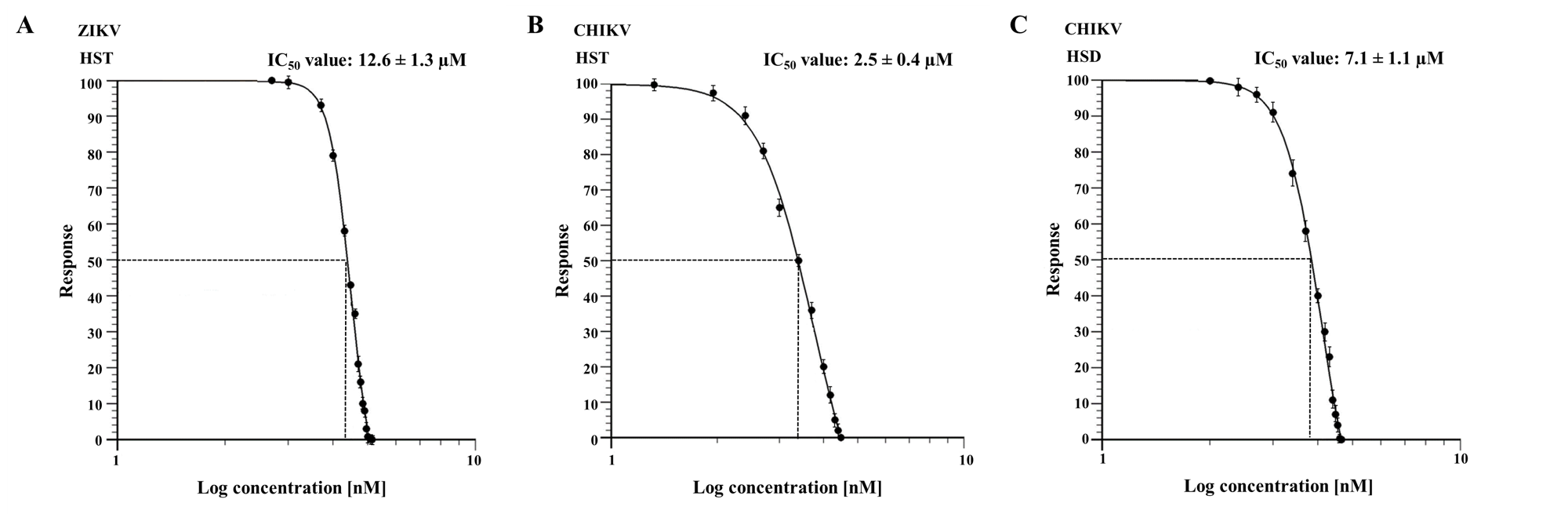

Supplement: S3 Fig — Dose response curve for (A and B) HST and (C) HSD. Half maximum inhibitory concentration (IC50) values were determined by nonlinear regression using 20 µM substrate (ZIKV NS2B/NS3pro), 3 µM substrate (CHIKV nsP2pro), 3 nM ZIKV NS2B/NS3pro, 1 µM CHIKV nsP2pro, with varying concentrations of the inhibitors. Data shown are the means ± SD from three independent measurements (n = 3). S1 Data contain the underlying data for the IC50 value determination. (TIF) [file pone.0246319.s003.tif]

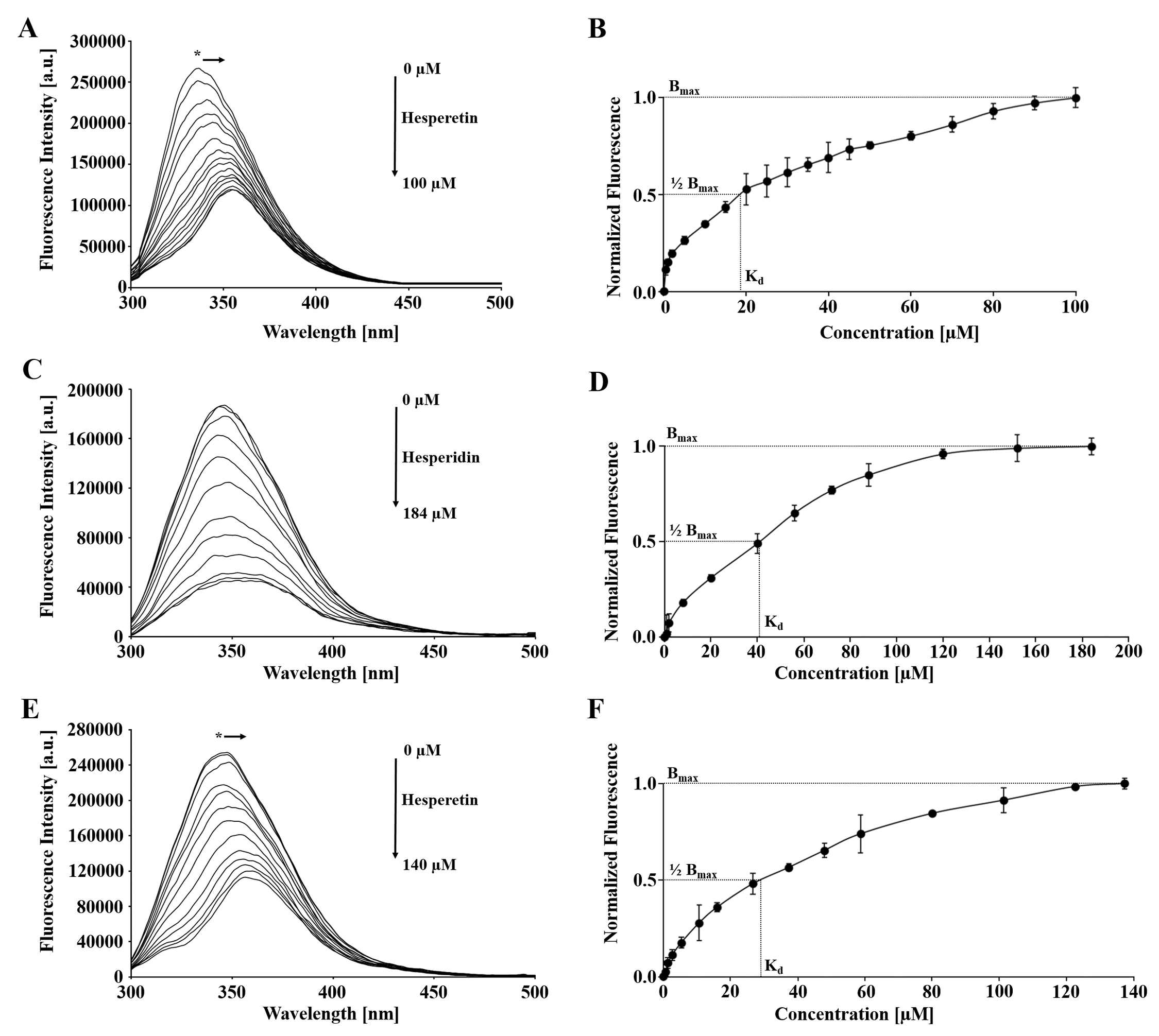

Supplement: S4 Fig — HST and HSD titration experiments. Data shown are the means ± SD from three independent measurements (n = 3). A: Fluorescence of ZIKV NS2B/NS3pro under influence of HST titration demonstrated a red excitation shift of visible Trp (*). B: Binding saturation curve and modified Hill equation determined a KD value of 17.8 ± 2.9 µM for the ZIKV NS2B/NS3pro-HST interaction. C: Fluorescence of CHIKV nsP2pro under influence of HSD titration. D: Binding saturation curve and modified Hill equation determined a KD value of 40.7 ± 2.0 µM for the CHIKV nsP2pro-HSD interaction. E: Fluorescence of CHIKV nsP2pro under influence of HST titration demonstrated a red excitation shift of visible Trp (*). F: Binding saturation curve and modified Hill equation determined a KD value of 31.6 ± 2.5 µM for the CHIKV nsP2pro-HST interaction. (TIF) [file pone.0246319.s004.tif]

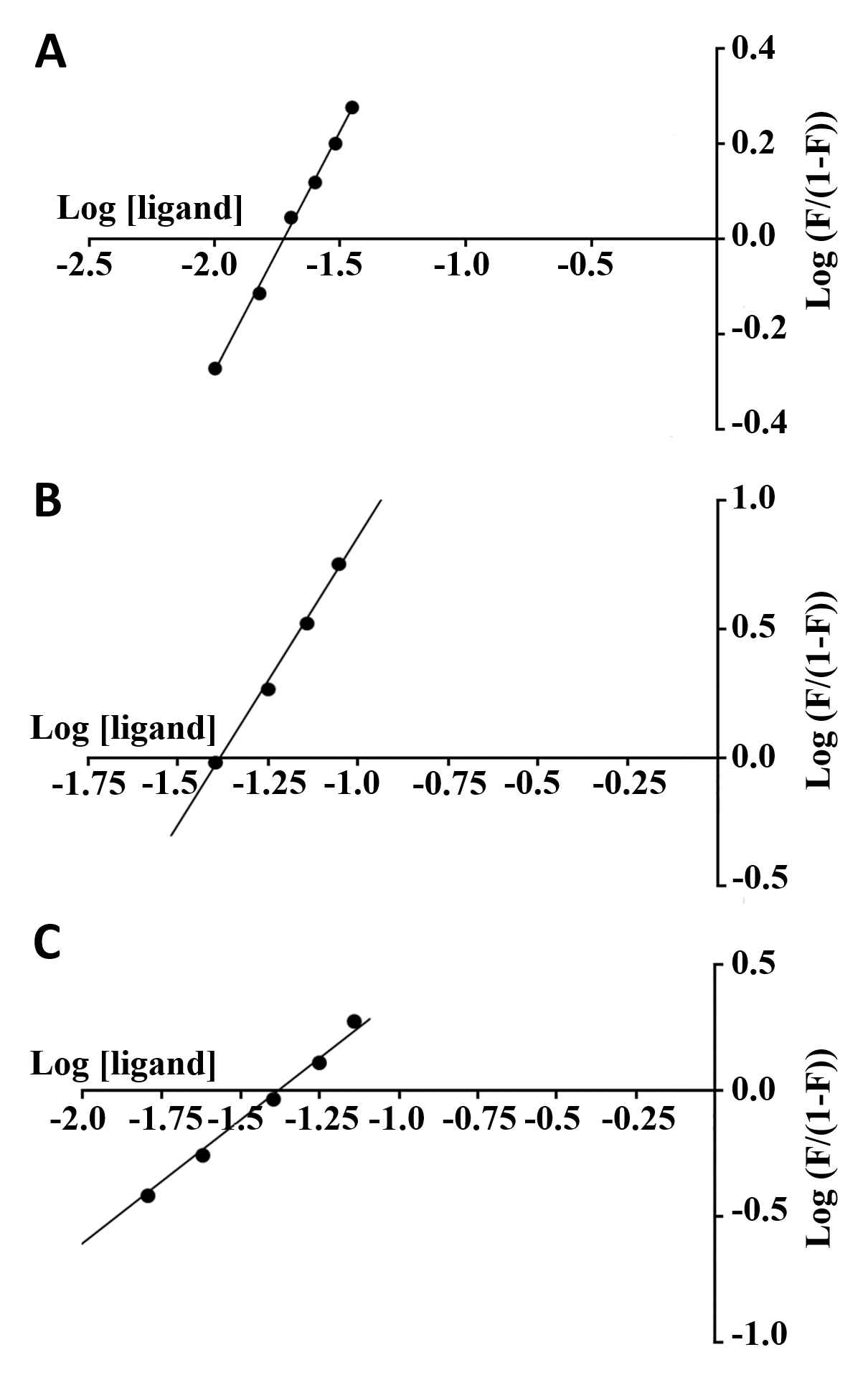

Supplement: S5 Fig — Based on fluorescence spectroscopy of Trp at 295 nm of ZIKV NS2B/NS3pro and CHIKV nsP2pro in the presence ligands. Intersection with x-axis corresponds to the logarithmic value of the KD. A: ZIKV NS2B/NS3pro-HST interaction. B: CHIKV nsP2pro-HSD interaction. C: CHIKV nsP2pro-HST interaction. (TIF) [file pone.0246319.s005.tif]

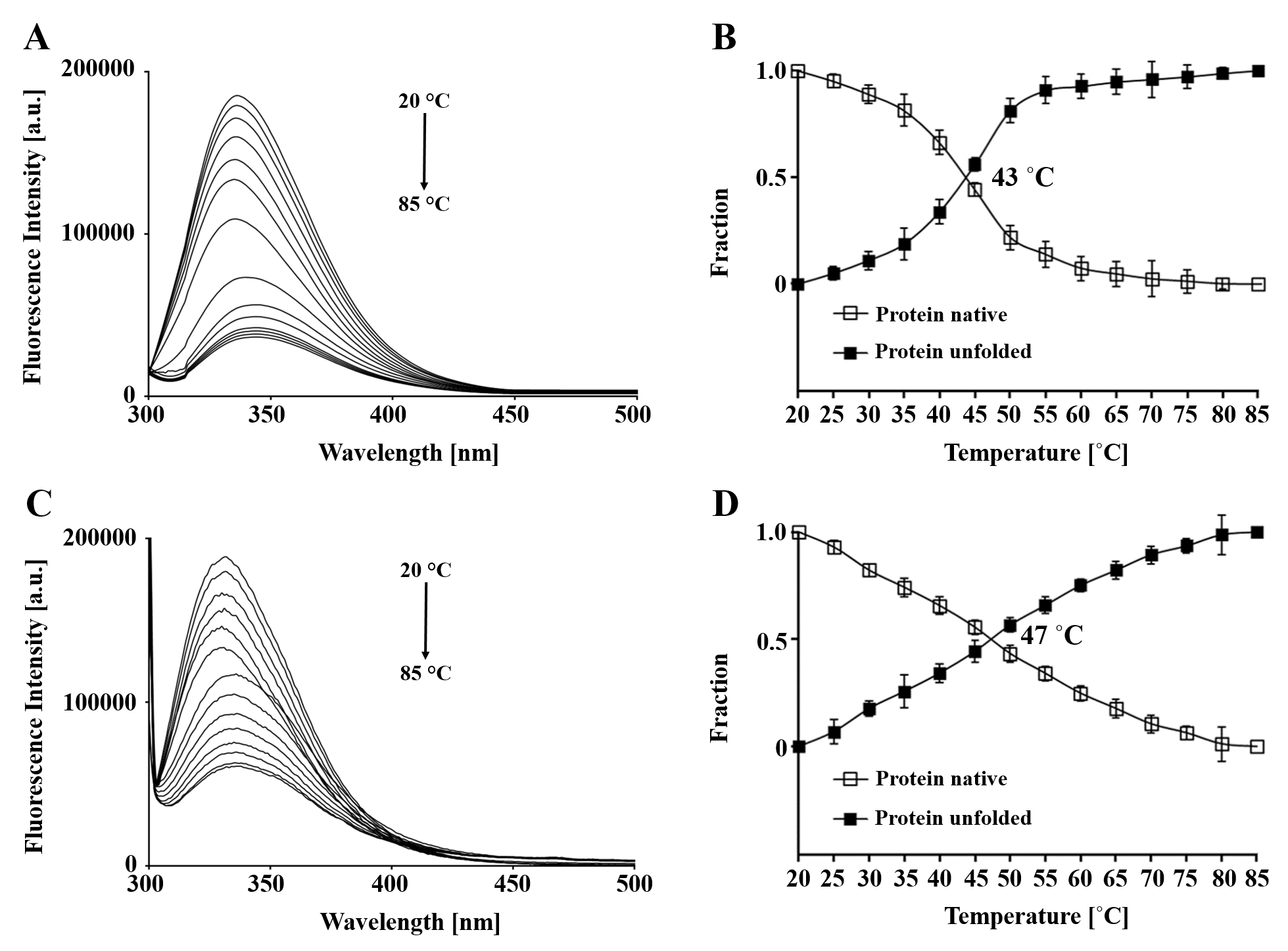

Supplement: S6 Fig — Data shown are the means ± SD from three independent measurements (n = 3). A: Fluorescence spectra during thermal denaturation of ZIKV NS2B/NS3pro. B: Plot of the native protein fraction (fN) and the unfolding protein fraction (fU), during thermal denaturation from 20 to 85°C. With increasing temperature fN decrease and fU increase, on the intersection of both curves the melting temperature (Tm) of 43°C was determined for ZIKV NS2B/NS3pro. C: Fluorescence spectra during thermal denaturation of CHIKV nsP2pro. D: Plot of the native protein fraction (fN) and the unfolding protein fraction (fU), during thermal denaturation from 20 to 85°C. The melting temperature (Tm) of 47°C was determined for CHIKV nsP2pro. (TIF) [file pone.0246319.s006.tif]

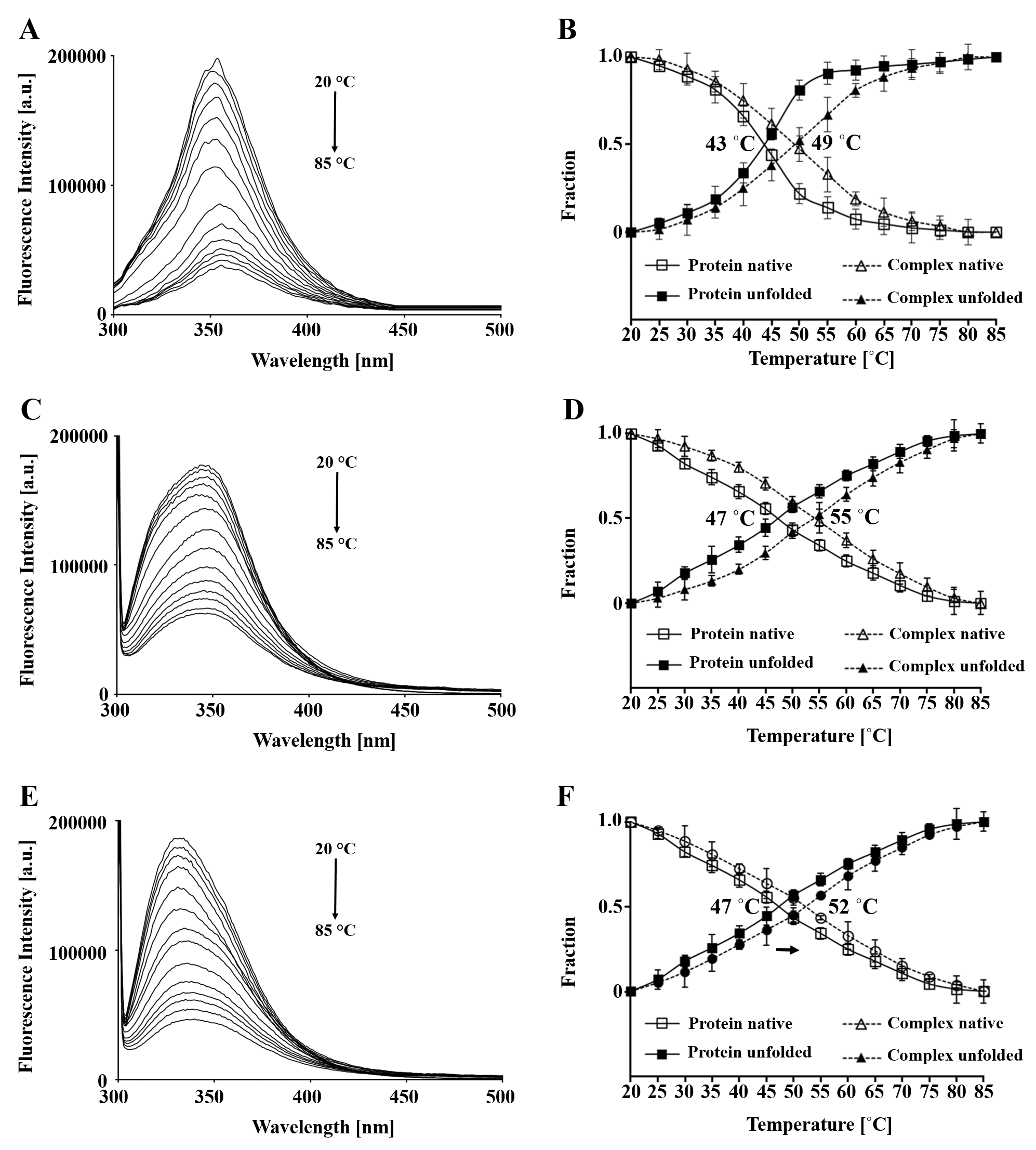

Supplement: S7 Fig — Data shown are the means ± SD from three independent measurements (n = 3). A: Fluorescence spectra during thermal denaturation of ZIKV NS2B/NS3pro-HST complex. B: Plot of the native protein fraction (fN) and the unfolding protein fraction (fU), during thermal denaturation from 20 to 85°C. The melting temperature (Tm) of 43°C was determined for ZIKV NS2B/NS3pro and for the ZIKV NS2B/NS3pro-HST complex the Tm increased to 49°C. C: Fluorescence spectra during thermal denaturation of CHIKV nsP2pro-HST complex. D: Plot of the native protein fraction (fN) and the unfolding protein fraction (fU), during thermal denaturation from 20 to 85°C. The melting temperature (Tm) of 47°C was determined for CHIKV nsP2pro and for the CHIKV nsP2pro-HST complex the Tm increased to 55°C. E: Fluorescence spectra during thermal denaturation of CHIKV nsP2pro-HSD complex. F: Plot of the native protein fraction (fN) and the unfolding protein fraction (fU), during thermal denaturation from 20 to 85°C. The melting temperature (Tm) of 43°C was determined for CHIKV nsP2pro and for the CHIKV nsP2pro-HSD complex the Tm changed to 52°C. (TIF) [file pone.0246319.s007.tif]

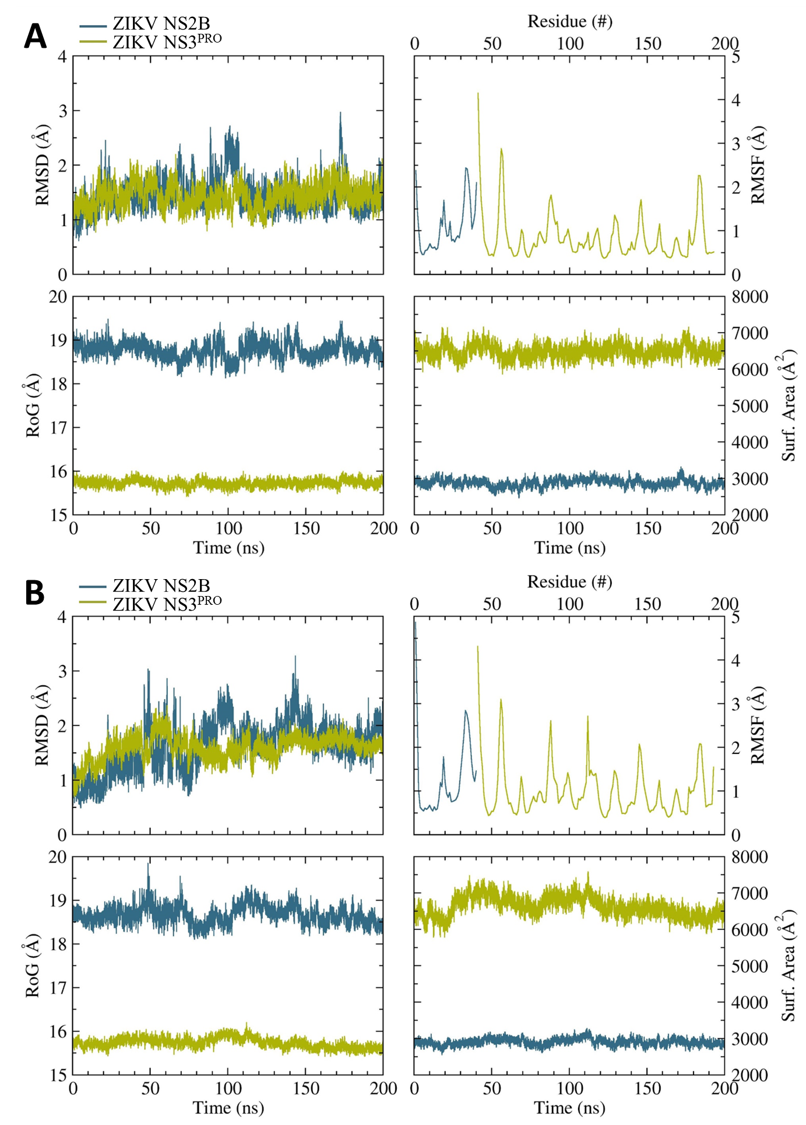

Supplement: S8 Fig — RMSD, RMSF, RoG and surface area changes over 200 ns of two independent MD runs. ZIKV NS2B (blue) and ZIKV NS3pro (green). The ZIKV NS2B cofactor is a small loop and is very flexible, which explain the bigger RoG and smaller surface area compared with NS3pro. A: MD run1, B: MD run2. (TIF) [file pone.0246319.s008.tif]

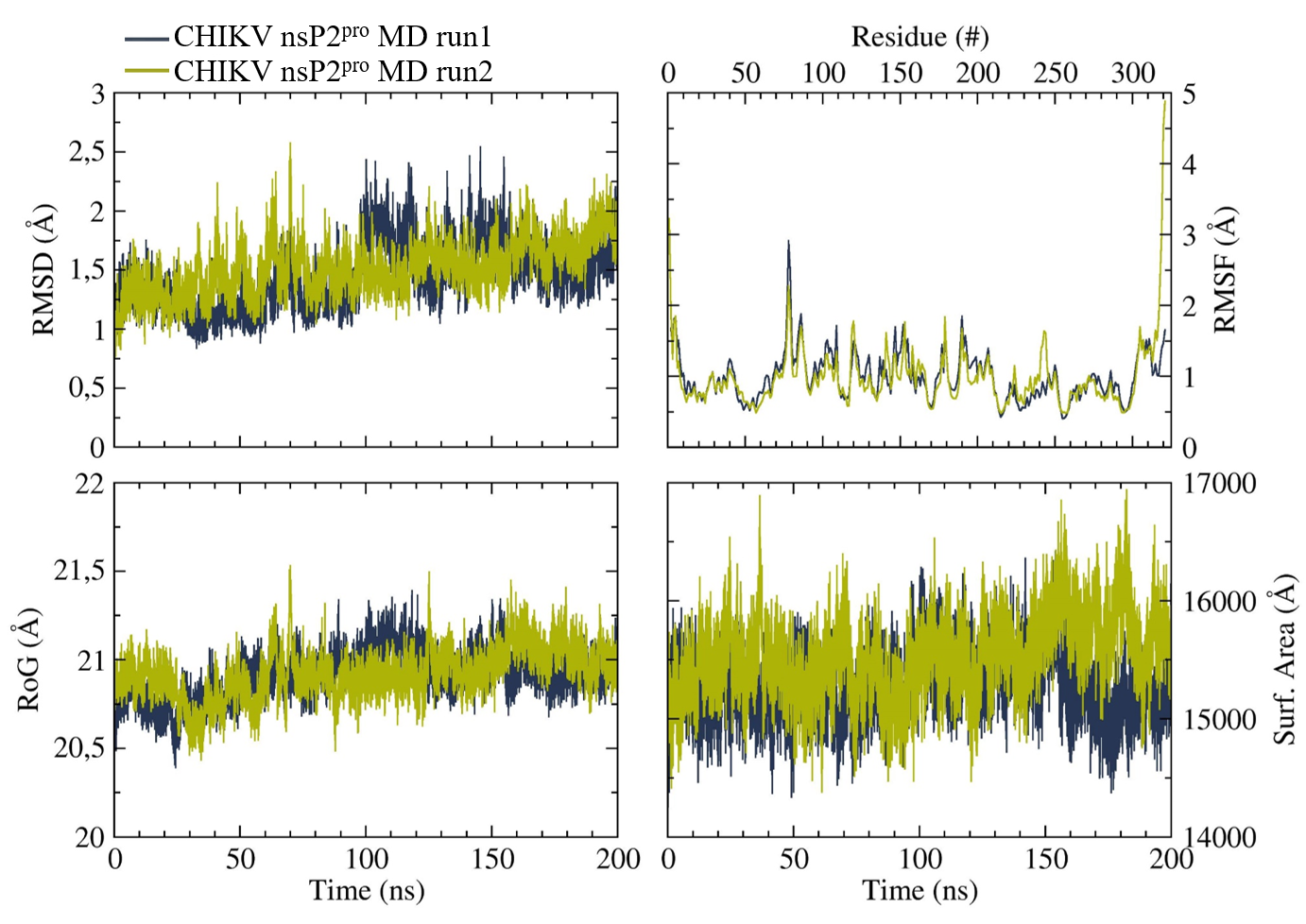

Supplement: S9 Fig — RMSD, RMSF, RoG and surface area changes over 200 ns of two independent MD runs. CHIKV nsP2pro MD run1 (dark blue) and CHIKV nsP2pro MD run2 (green). (TIF) [file pone.0246319.s009.tif]

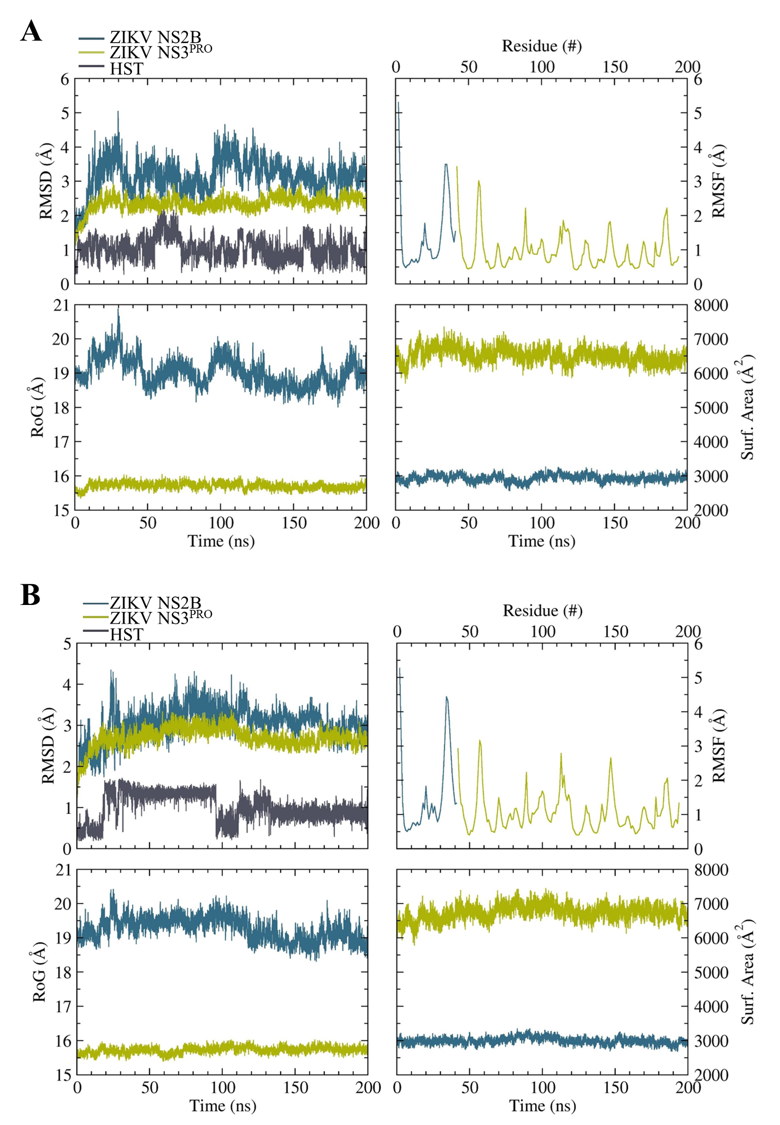

Supplement: S10 Fig — RMSD, RMSF, RoG and surface area changes over 200 ns of two independent MD runs. ZIKV NS2B (blue), ZIKV NS3pro (green) and HST (black). RMSD as function of time. A: ZIKV NS2B/NS3pro-HST MD run1. B: ZIKV NS2B/NS3pro-HST MD run2. (TIF) [file pone.0246319.s010.tif]

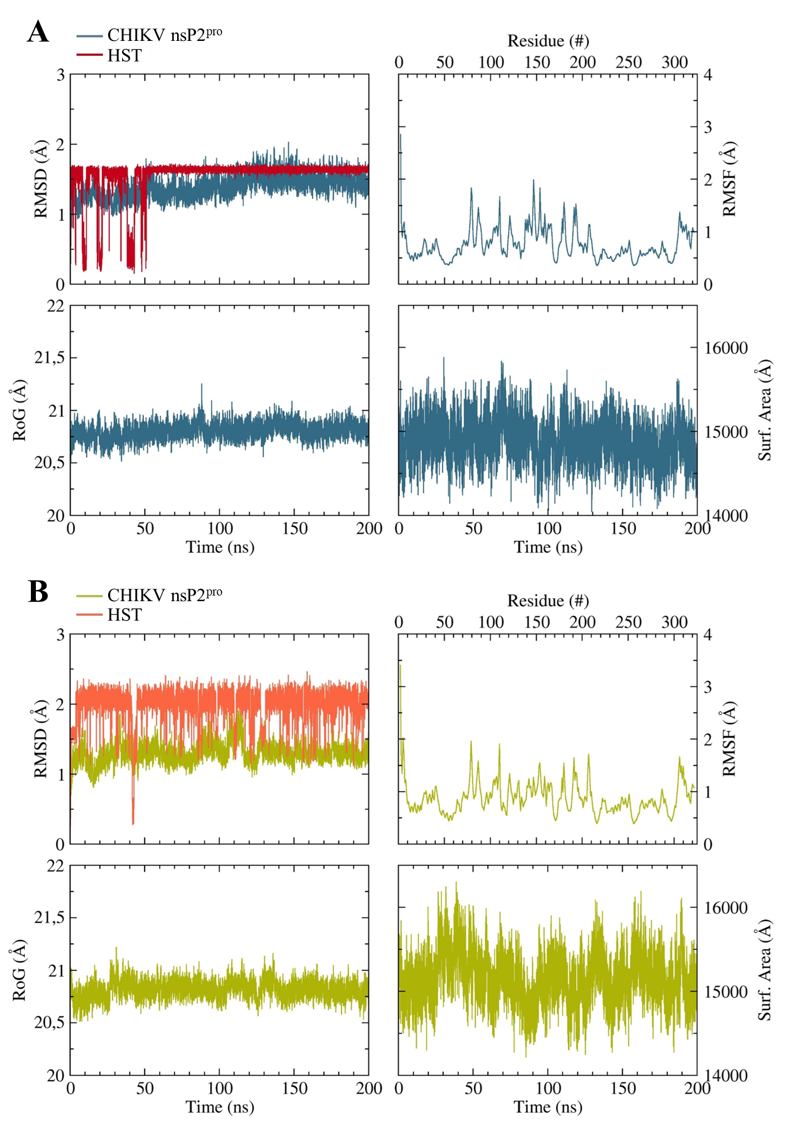

Supplement: S11 Fig — RMSD, RMSF, RoG and surface area changes over 200 ns of two independent MD runs. RMSD as function of time. A: CHIKV nsP2pro-HST MD run1, CHIKV nsP2pro (dark blue) and HST (red). B: CHIKV nsP2pro-HST MD run2, CHIKV nsP2pro (green) and HST (pink). (TIF) [file pone.0246319.s011.tif]

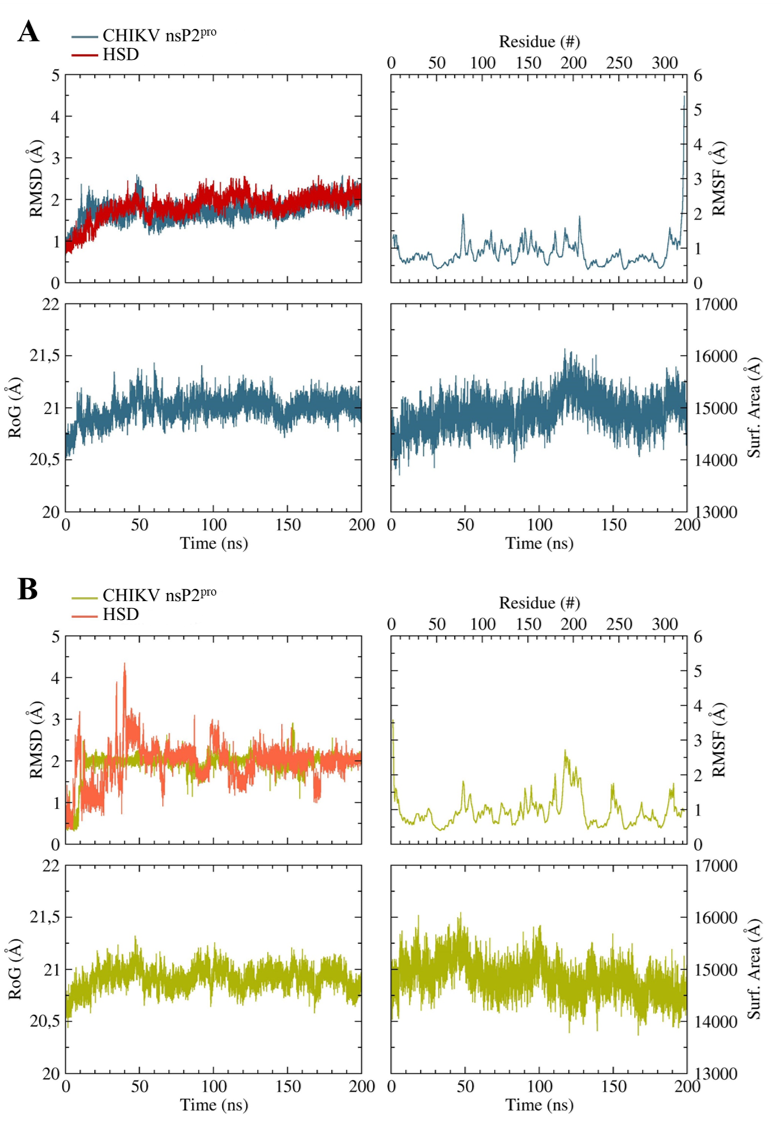

Supplement: S12 Fig — RMSD, RMSF, RoG and surface area changes over 200 ns of two independent MD runs. RMSD as function of time. A: CHIKV nsP2pro-HSD MD run1, CHIKV nsP2pro (dark blue) and HSD (red). B: CHIKV nsP2pro-HSD MD run2, CHIKV nsP2pro (green) and HSD (pink). (TIF) [file pone.0246319.s012.tif]

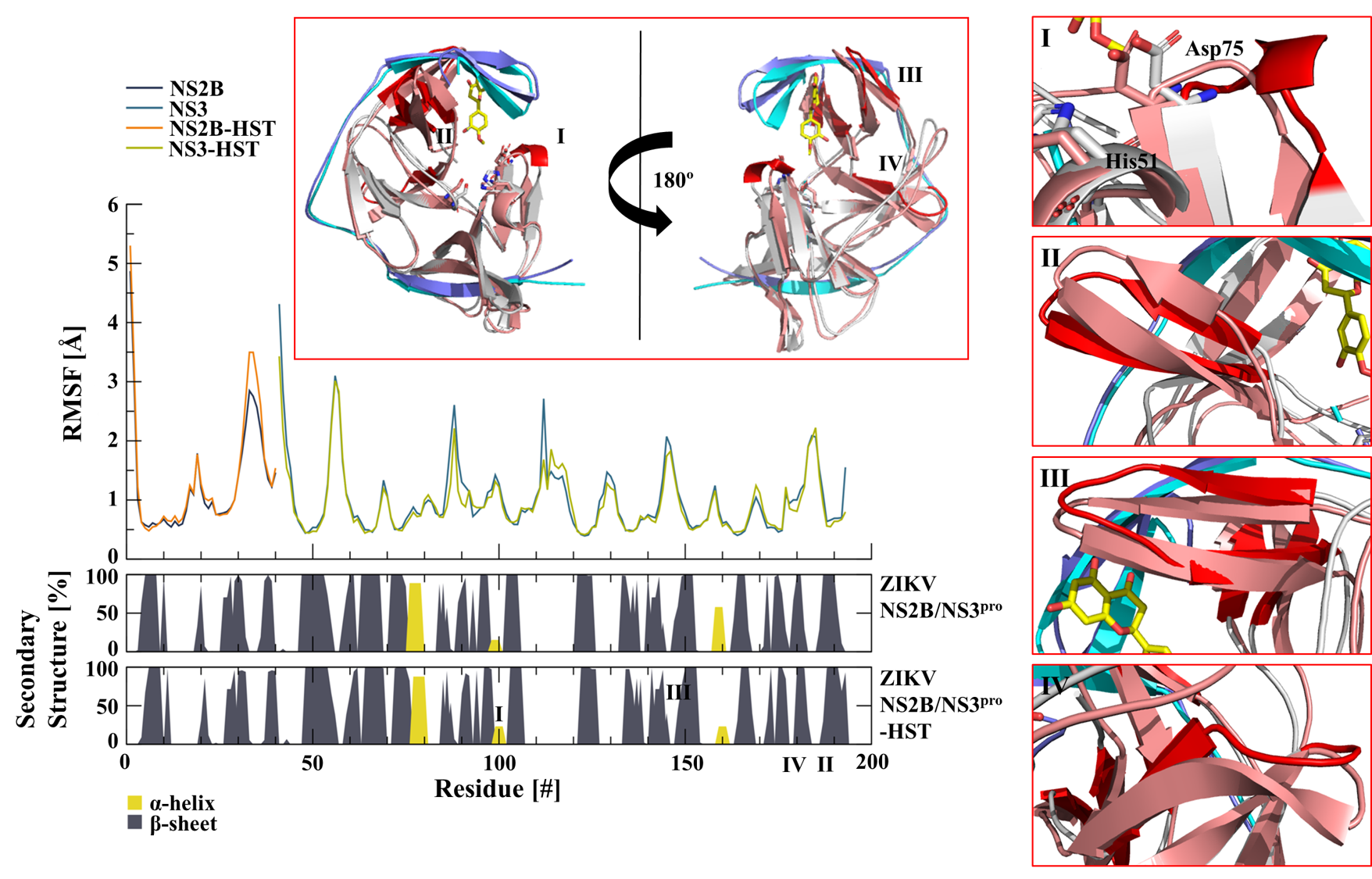

Supplement: S13 Fig — ZIKV NS2B/NS3pro: NS3pro in rosé, NS2B in cyan, ZIKV NS2B/NS3pro-HST: NS3pro in gray, NS2B in blue and HST in yellow. Secondary structure changes highlighted in red. A: Overlay of the RMSF of ZIKV NS2B/NS3pro with and without HST. B: Secondary structure changes over 200 ns of ZIKV NS2B/NS3pro with and without HST. I-IV labels small changes in the secondary structure. C: Structural overlay of ZIKV NS2B/NS3pro with and without HST. Secondary structure changes highlighted in red and the number code. Right panel: Zoom view on the secondary structure changes. (TIF) [file pone.0246319.s013.tif]

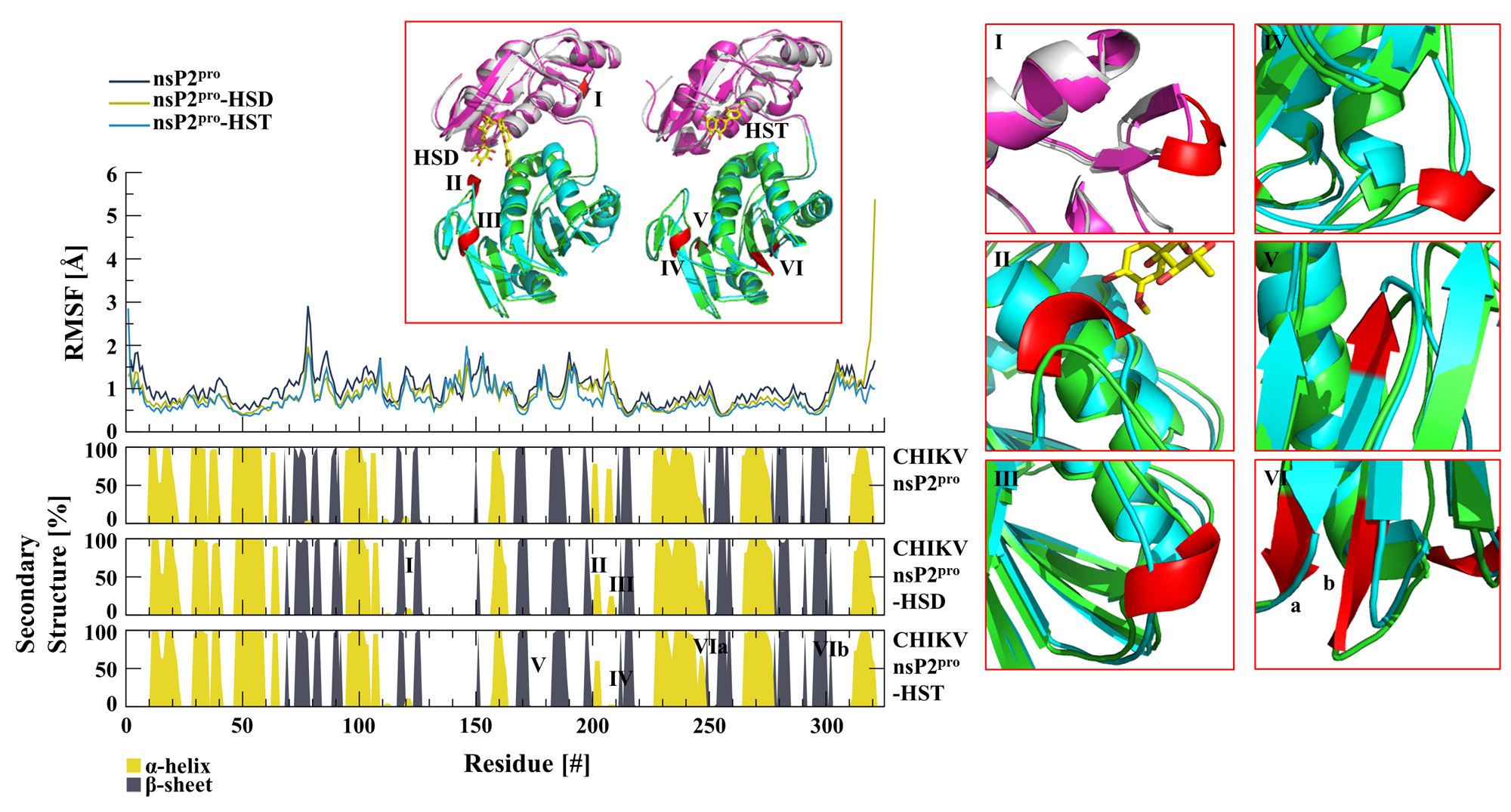

Supplement: S14 Fig — CHIKV nsP2pro: protease domain in violet, methyl transferase domain in cyan, CHIKV nsP2proHST/HSD: protease domain in gray, methyl transferase domain in green, HST/HSD in yellow. A: Overlay of the RMSF of CHIKV nsP2pro with and without HSD and HST. B: Secondary structure changes over 200 ns of CHIKV nsP2pro with and without HSD and HST. I-VI labels small changes in the secondary structure. C: Structural overlay of CHIKV nsP2pro with and without HSD and HST. Secondary structure changes highlighted in red and the number code. Right panel: Zoom view on the secondary structure changes. (TIF) [file pone.0246319.s014.tif]

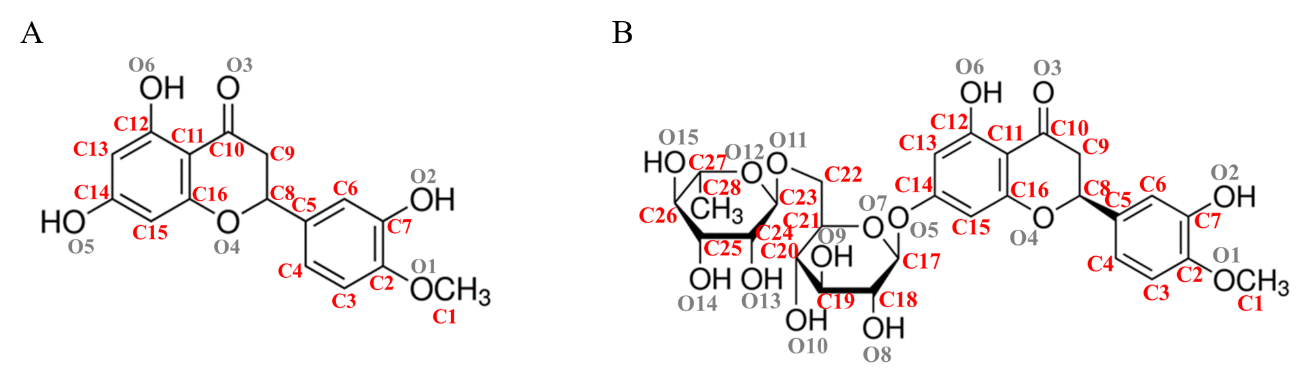

Supplement: S15 Fig — A: HST and B: HSD. (TIF) [file pone.0246319.s015.tif]

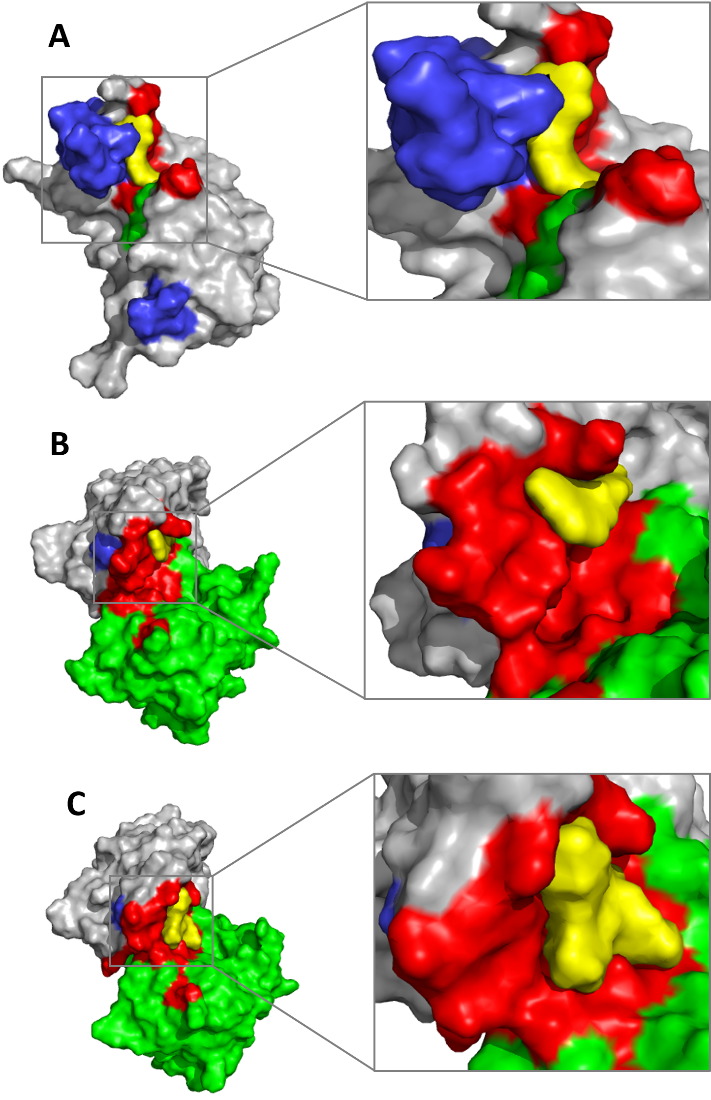

Supplement: S16 Fig — Surface view of the protease structures and the ligands. A: ZIKV NS2B/NS3pro-HST complex. NS2B is colored in blue, NS3pro in gray, HST in yellow, active site in green and allosteric site in red. B: CHIKV nsP2pro-HST complex. Protease domain is colored in gray, methyltransferase domain in green, HST in yellow, active site in blue and allosteric site in red. C: CHIKV nsP2pro-HSD complex. Protease domain is colored in gray, methyltransferase domain in green, HSD in yellow, active site in blue and allosteric site in red. (TIF) [file pone.0246319.s016.tif]

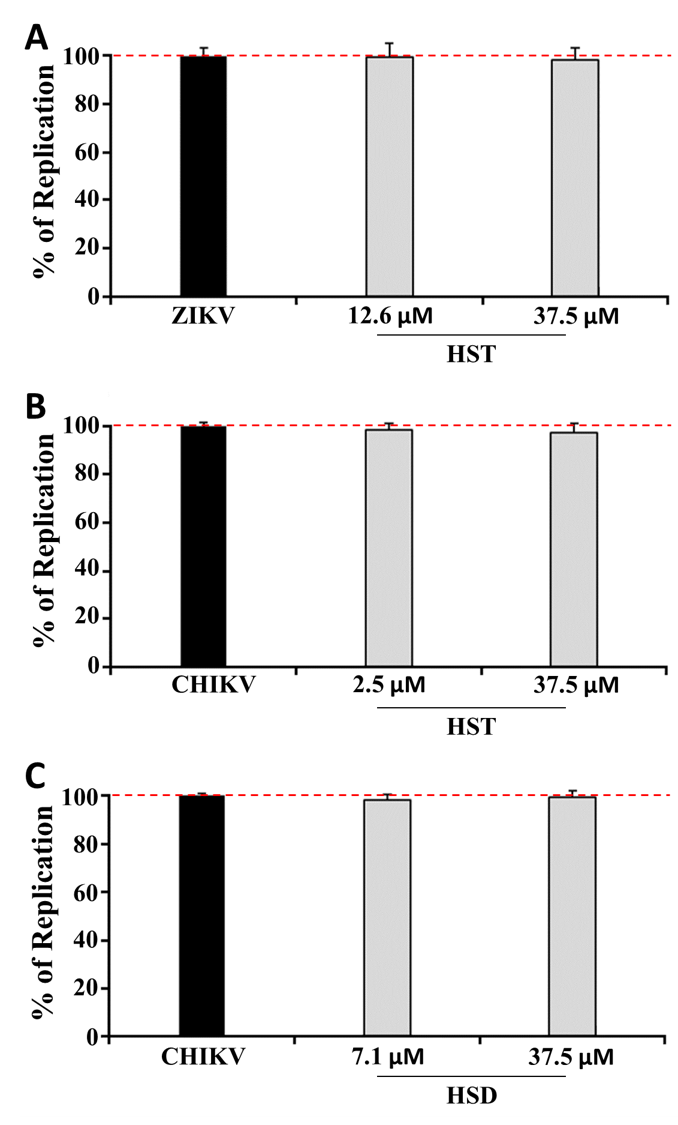

Supplement: S17 Fig — Data shown are the means ± SD from three independent measurements (n = 3). There were no antiviral activities of HST and HSD observable. Virus control (black) and two inhibitor concentrations (grey). A: ZIKV under HST influence. B: CHIKV under HST influence and C: CHIKV under HSD influence. (TIF) [file pone.0246319.s017.tif]
