# Supplementary material for: In vitro study of Hesperetin and Hesperidin as inhibitors of zika and chikungunya virus proteases
Source: PLoS One. 2021 Mar 4;16(3):e0246319. doi: 10.1371/journal.pone.0246319 (PMC7932080; doi:10.1371/journal.pone.0246319)
Supplement: S1 Table — (DOCX) [file pone.0246319.s018.docx]

**S1 Table.** Drugs or inhibitors affecting flavivirus and alphavirus proteases or other virus target proteins.

| Compound | ZIKV NS2B/NS3 | DENV NS2B/NS3 | CHIKV NSP2 | Other targets | references |
| --- | --- | --- | --- | --- | --- |
| Drug | | | | | |
| Nelfinavir | - |  |  |  | [1] |
| Bromocriptine |  | - | - | DENV replication | [2]  [3] |
| Policresulen | - |  | - |  | [4] |
| Lopinavir-ritonavir |  | - | - |  | [5] |
| Hydroxychloroquine |  | - | - | DENV, CHIKV internalization | [6-8] |
| Flavanone | | | | | |
| Hesperetin | - | - | - | CHIKV replication | [9] |
| Naringenin | - | - | - | CHIKV and DENV replication | [9,10] |
| Quinone | | | | | |
| Anthraquinone | - |  | - |  | [11] |
| Antibiotic | | | | | |
| Novobiocin |  | - | - |  | [5] |
| Natural alkaloid | | | | | |
| Harringtonine | - | - |  |  | [12] |
| Terpenoid | | | | | |
| Prostratin | - | - |  |  | [13] |
| Tetrapyrrole | | | | | |
| Biliverdin |  |  | - | ZIKV NS2B/NS3^pro^  suggested target | [14,15] |
| Thiazolidine | | | | | |
| Thiazolidinone derivatives | - |  | - | CHIKV NsP2^pro^  suggested target | [16,17] |
| Protein and peptide inhibitors | | | | | |
| Aprotinin |  |  | - | Serine protease inhibitor | [18,19] |
| Peptidylamids |  |  | - |  | [20] |
| Peptidylboronic acids |  |  | - |  | [20] |

**References**

1. Soumendranath B, Delang L, Kaptein S, Neyts J, Leyssen P, Jayaprakash V. Reaching beyond HIV/HCV: nelfinavir as a potential starting point for broad-spectrum protease inhibitors against dengue and chikungunya virus. RSC Adv. 2015;5:85938-85949. <https://doi.org/10.1039/C5RA14469H>
2. Kato F, Ishida Y, Oishi S, Fujii N, Watanabe S, Vasudevan SG, et al. Novel antiviral activity of bromocriptine against dengue virus replication. Antiviral Res. 2016,131:141-147. <https://doi.org/10.1016/j.antiviral.2016.04.014>
3. Chan JFW, Chik KKH, Yuan S, Yip CC, Zhu Z, Tee KM, et al. Novel Antiviral Activity and Mechanism of Bromocriptine as a Zika Virus NS2B-NS3 Protease Inhibitor. Antiviral Res. 2017;141:29-37. <https://doi.org/10.1016/j.antiviral.2017.02.002>
4. Wu DW, Mao F, Ye Y, Yu CY, Tsai WL, Chen JJ, et al. Policresulen, a novel NS2B/NS3 protease inhibitor, effectively inhibits the replication of DENV2 virus in BHK-21 cells. Acta Pharmacol. Sin. 2015;9:1126-1136. <https://doi.org/10.1038/aps.2015.56>
5. Yuan S, Chan JFW, Den-Haan H, Chik KK, Zhang AJ, Chan CC, et al. Structure-Based Discovery of Clinically Approved Drugs as Zika Virus NS2B-NS3 Protease Inhibitors That Potently Inhibit Zika Virus Infection *In Vitro* and *In Vivo*. Antiviral Res. 2017;145:33-43. <https://doi.org/10.1016/j.antiviral.2017.07.007>
6. Khan M, Santhosh SR, Tiwari M, Lakshmana Rao PV, Parida M. Assessment of *in vitro* prophylactic and therapeutic efficacy of chloroquine against Chikungunya virus in vero cells. J. Med. Virol. 2010;82:817–824. <https://doi.org/10.1002/jmv.21663>
7. Wang LF, Lin YS, Huang NC, Yu CY, Tsai WL, Chen JJ, et al. Hydroxychloroquine-inhibited dengue virus is associated with host defense machinery. J. Interferon Cytokine Res. 2015;35:143-156. <https://doi.org/10.1089/jir.2014.0038>
8. Kumar A, Liang B, Aarthy M, Singh SK, Garg N, Mysorekar IU, et al. Hydroxychloroquine inhibits Zika virus NS2B-NS3 protease. ACS omega. 2018;3:18132-18141. <https://doi.org/10.1021/acsomega.8b01002>
9. Ahmadi A, Hassandarvish P, Lani R, Yadollahi P, Jokar A, AbuBakar S, et al. Inhibition of chikungunya virus replication by hesperetin and naringenin. RSC Adv. 2016;6:69421-69430. <https://doi.org/10.1039/C6RA16640G>
10. Zandi K, Teoh B, Sam S, Wong P, Mustafa MR, AbuBakar A. *In vitro* antiviral activity of Fisetin, Rutin and Naringenin against Dengue virus type-2. J. Med. Plants Res. 2011;55534-5539.
11. Tomlinson SM, Malmstrom RD, Russo A, Mueller N, Pang YP, Watowich SJ. Structure-based discovery of dengue virus protease inhibitors. Antiviral Res. 2009;82:110-114. <https://doi.org/10.1016/j.antiviral.2009.02.190>
12. Kaur P, Thiruchelvan M, Lee RCH, Chen H, Chen KC, Ng ML, et al. Inhibition of chikungunya virus replication by harringtonine, a novel antiviral that suppresses viral protein expression. Antimicrob. Agents Chemother. 2013;57:155-167. <https://doi.org/10.1128/AAC.01467-12>
13. Bourjot M, Delang L, Nguyen VH, Neyts J, Guéritte F, Leyssen P, et al. Prostratin and 12-O-tetradecanoylphorbol 13-acetate are potent and selective inhibitors of chikungunya virus replication. J. Nat. Prod. 2012;75:2183-2187. <https://doi.org/10.1021/np300637t>
14. Tseng CK, Lin CK, Wu YH, Chen YH, Chen WC, Young KC, et al. Human heme oxygenase 1 is a potential host cell factor against dengue virus replication. Sci Rep. 2016;6:32176. <https://doi.org/10.1038/srep32176>
15. El Kalamouni C, Frumence E, Bos S, Turpin J, Nativel B, Harrabi W, et al. Subversion of the Heme Oxygenase-1 Antiviral Activity by Zika Virus. Viruses 2019;11:E2. <https://doi.org/10.3390/v11010002>
16. Yusufzai SK, Osman H, Khan MS, Abd Razik BM, Ezzat MO, Mohamad S, et al. 4-Thiazolidinone coumarin derivatives as two-component NS2B/NS3 DENV flavivirus serine protease inhibitors: synthesis, molecular docking, biological evaluation and structure–activity relationship studies. Chem. Cent. J. 2018;12:69. <https://doi.org/10.1186/s13065-018-0435-0>
17. Jadav SS, Sinha BN, Hilgenfeld R, Pastorino B, de Lamballerie X, Jayaprakash V. Thiazolidone derivatives as inhibitors of chikungunya virus. Eur. J. Med. Chem. 2015;89:172-178. <https://doi.org/10.1016/j.ejmech.2014.10.042>
18. Chen X, Yang K, Wu C, Chen C, Hu C, Buzovetsky O, et al. Mechanisms of activation and inhibition of Zika virus NS2B-NS3 protease. Cell Res. 2016;26:1260-1263. <https://doi.org/10.1038/cr.2016.116>
19. Lin KH, Ali A, Rusere L, Soumana DI, Yilmaz NK, Schiffer CA. Dengue virus NS2B/NS3 protease inhibitors exploiting the prime side. J. Virol. 2017;91:e00045-17. <https://doi.org/10.1128/JVI.00045-17>
20. Nitsche C, Zhang L, Weigel LF, Schilz J, Graf D, Bartenschlager R, et al. Peptide–boronic acid inhibitors of flaviviral proteases: medicinal chemistry and structural biology. J. Med. Chem. 2016;60:511-516. <https://doi.org/10.1021/acs.jmedchem.6b01021>
