# Supplementary material for: In vitro study of Hesperetin and Hesperidin as inhibitors of zika and chikungunya virus proteases
Source: PLoS One. 2021 Mar 4;16(3):e0246319. doi: 10.1371/journal.pone.0246319 (PMC7932080; doi:10.1371/journal.pone.0246319)
Supplement: S2 Table — (DOCX) [file pone.0246319.s019.docx]

**S2 Table.** Contributing amino acids in the Substrate-binding sites of

ZIKV NS2B/NS3^pro^ and CHIKV nsP2^pro^.

| Substrate subsite* | ZIKV NS2B/NS3^pro^  residues | CHIKV nsP2^pro^ residues |
| --- | --- | --- |
| S1` | Val36 | Ala8 |
|  | His51 | Asn9 |
|  | Ser135 | Cys11 |
|  |  | Lys14 |
|  |  | His81 |
| S1 | Tyr150 | Asn9 |
|  | Ser163 | Cys11 |
|  | Ile165 | Trp12 |
|  |  | Asn80 |
|  |  | His81 |
| S2 | His51 | Trp12 |
|  | Gly151 | Tyr45 |
|  | Asn152 | Trp82 |
| S3 | Leu128 | Tyr77 |
|  | Asp129 | Asn80 |
|  | Ala132 | Met240 |
|  | Val155 |  |
| S4 | Gly153 | Asn80 |
|  | Val154 | Trp82 |
|  | Val155 | Gln239 |
|  |  | Asp244 |
| Oxyanion hole | Ala132 | Ala8 |
|  | Gly133 | Asn9 |
|  | Thr134 | Val10 |
|  | Ser135 | Cys11 |

*[1, 2]

**References**

1. Narwal M, Singh H, Pratap S. Crystal structure of chikungunya virus nsP2 cysteine protease reveals a putative flexible loop blocking its active site. Int. J. Biol. Macromol. 2018;116:451-462. <https://doi.org/10.1016/j.ijbiomac.2018.05.007>
2. Phoo WW, Li Y, Zhang Z, Lee MY, Loh YR, Tan YB, et al. Structure of the NS2B-NS3 protease from Zika virus after self-cleavage. Nat. Commun. 2016;7:1-8. <https://doi.org/10.1038/ncomms13410>
