# Supplementary material for: In vitro study of Hesperetin and Hesperidin as inhibitors of zika and chikungunya virus proteases
Source: PLoS One. 2021 Mar 4;16(3):e0246319. doi: 10.1371/journal.pone.0246319 (PMC7932080; doi:10.1371/journal.pone.0246319)
Supplement: Attachment — Submitted filename: Eberle et al Plos one review.docx [file pone.0246319.s021.docx]

The study by Erbele et al., investigates the capacity of two natural plant-based compounds (HSD and HST) to inhibit the protease activity of ZIKV NS2B/NS3pro and CHIKV NSP2pro *in vitro*. The authors demonstrate inhibitory effects of both compounds against the CHIKV protease while only HST displays a modest inhibitory effect against the ZIKV protease. Through a series of dose response experiments, fluorescence spectroscopy experiments, thermal stability assays, as well as *in-silico* molecular docking analyses, the authors define the IC50 values of their compounds in their *in vitro* protease activity assays and propose the inhibitors to act non-competitively by binding allosterically to both viral proteases, thus inducing a conformational change which the authors propose alters protease activity. For the most part the biochemical data are convincing, but with all purely biochemical studies, the biological relevance of the findings is unclear. Although these compounds have been shown to possess antiviral activity against CHIKV, whether they possess antiviral activity against ZIKV is unknown. The authors should attempt to demonstrate the antiviral activity of their compounds to support their proposed usage of HSD and HST as therapeutics to treat ZIKV and CHIKV infection/co-infection. The manuscript would also benefit from the addition of a few specific controls. Specific points for the authors to address are below:

1. The authors should test the antiviral activity of HSD and HST against ZIKV infection *in vitro.* It would also be useful to show the antiviral activity of these compounds against CHIKV infection in the authors hands but this is less critical as already shown by others.
2. It seems as if the binding affinities of HST and HSD for CHIKV and ZIKV proteases do not correlate with their inhibitor capacity against protease activity. Could the other comment on this observation?
3. Since the authors propose usage of HSD/HST or modified compounds, to treat ZIKV and CHIKV infected patients, some discussion on the potential maximal tolerated dose, pharmacological kinetics, and dose of administration should be added to the manuscript.
4. Do the authors hypothesize the anti-protease activity HSD and HST to be specific to these viral proteases or much broader? Since HST was previously shown to inhibit protease activity of snake venom, perhaps the activity is quite broad? The authors should discuss the potential for off-target effects of these compounds and how this would be tolerated by cells in vitro or human patients.
5. It would be useful for the authors to measure cytotoxicity levels of HSD and HST on relevant human cell *in vitro* using the compound doses tested in this paper.
6. Statistics should be added to figure 2.

Minor Comments:

1. Line 103/104: should be written to treat co-infections?
2. Some information about the strains the CHIKV and ZIKV recombinant proteins come from should be provided in the methods.
3. Figure 2: For ZIKV protease inhibition what is the % activity observed required to call a compound a hit. A positive and negative control compound would be helpful to interpret the results.
